# Therapeutic options for different metastatic sites arising from renal cell carcinoma: A review

**DOI:** 10.1097/MD.0000000000038268

**Published:** 2024-05-24

**Authors:** Xue Wang, Lin Qian, Zengxing Qian, Qihang Wu, Dongying Cheng, Junjun Wei, Lingmin Song, Shuaihuai Huang, Xiaodong Chen, Ping Wang, Guobin Weng

**Affiliations:** a Zhejiang Key Laboratory of Pathophysiology, Ningbo University, Ningbo, China; b Department of Urologic Surgery, Ningbo Yinzhou No. 2 Hospital, Ningbo, China; c Department of Clinical Laboratory, Ningbo Yinzhou No. 2 Hospital, Ningbo, China; d Department of community, Ningbo Yinzhou No. 3 Hospital, Ningbo, China.

**Keywords:** clinical treatment, metastasis, renal cell carcinoma, stratify, the location of metastasis

## Abstract

Renal cell carcinoma (RCC) stands among the top 10 malignant neoplasms with the highest fatality rates. It exhibits pronounced heterogeneity and robust metastatic behavior. Patients with RCC may present with solitary or multiple metastatic lesions at various anatomical sites, and their prognoses are contingent upon the site of metastasis. When deliberating the optimal therapeutic approach for a patient, thorough evaluation of significant risk factors such as the feasibility of complete resection, the presence of oligometastases, and the patient’s functional and physical condition is imperative. Recognizing the nuanced differences in RCC metastasis to distinct organs proves advantageous in contemplating potential treatment modalities aimed at optimizing survival outcomes. Moreover, discerning the metastatic site holds promise for enhancing risk stratification in individuals with metastatic RCC. This review summarizes the recent data pertaining to the current status of different RCC metastatic sites and elucidates their role in informing clinical management strategies across diverse metastatic locales of RCC.

## 1. Introduction

Renal cell carcinoma (RCC) constitutes 95% of renal tumors and is among the most prevalent malignant neoplasms affecting the urinary system.^[1]^ In 2024, United States statistics reported 81,610 new cases of kidney cancer, ranking 6th among males and 8th among females, with 14,390 recorded deaths.^[2]^ The classic clinical triad of RCC, characterized by gross hematuria, pain, and palpable abdominal mass, is rare. Instead, it commonly presents with nonspecific systemic symptoms.^[3]^ Improved screening has led to a higher proportion of RCC diagnoses at early stages, facilitating curative interventions via radical surgical intervention.^[4]^ However, approximately 30% of patients experience metastasis following radical surgery.^[5]^ Advances in therapeutic strategies, including targeted drugs and immune checkpoint inhibitors (ICIs), have enhanced the prognosis of metastatic RCC (mRCC).^[6]^ Nonetheless, differences exist in metastasis principles, treatment strategies, and prognosis among various metastatic sites.^[7]^ Therefore, understanding risk stratification based on metastatic site, incorporating attributes of distinct metastatic sites, prognostic factors, and treatment approaches, is crucial for evaluating and predicting the therapeutic outcomes and prognosis of mRCC patients.

## 2. Metastatic RCC

**Figure 1.**
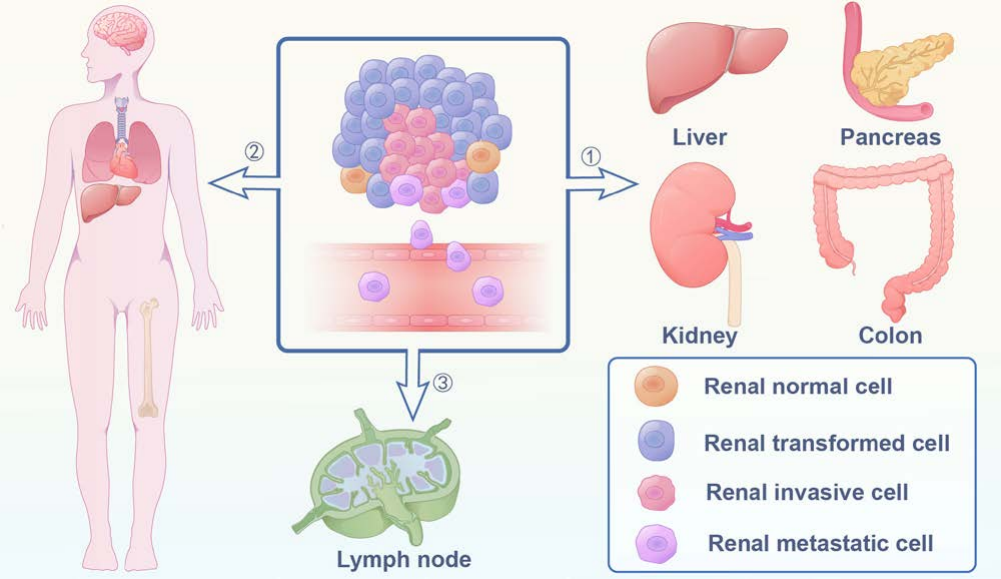
Pathway and site of renal cell carcinoma metastasis.

RCC is a highly heterogeneous tumor that exhibits significant resistance to conventional systemic therapies and a notable tendency for metastasis.^[8,9]^ Multiple organs possess the ability to accommodate metastatic cells originating from the kidney through 3 pathways: ① local infiltration, ② the hematogenous spread route, ③ the lymphatic route (Fig. 1).^[10]^ About 61% of mRCC patients develop solitary metastatic lesions, while 39% encounter multifocal metastatic disease, often with concurrent bone and brain metastases alongside metastases at other sites.^[11]^ The prognosis of RCC patients relies on the metastasis site.^[11]^ Notably, the survival duration of RCC patients with a single metastasis significantly exceeds that of those with multiple metastases.^[12]^ Additionally, younger patients tend to exhibit a higher frequency of multifocal metastatic disease.^[11]^

Despite changes in treatment strategies that have improved response rates and extended progression free survival (PFS) and overall survival (OS), patients with mRCC continue to face a challenging prognosis.^[13]^ Typically, the median survival of patients with mRCC ranges from 6 to 12 months, with a 5-year survival rate of <20%.^[14,15]^ Currently, 6 universally recognized prognostic risk factors are used to predict survival in patients with mRCC: Karnofsky Performance Status < 80%, hemoglobin level < lower normal level, time from diagnosis to systemic treatment < 1 year, corrected serum calcium > level > upper normal level (UNL), neutrophil count > UNL, and platelet count > UNL. Patients devoid of risk factors are anticipated to attain a median OS exceeding 43 months, whereas those with 1 or 2 risk factors experience an OS of approximately 23 months, and individuals with 3 or more risk factors demonstrate an OS of merely 8 months.^[16,17]^ To optimize the survival of mRCC patients, precise patient selection predicated on baseline prognostic factors and various aspects of the patient’s clinical status, including performance status, age, disease progression, site of metastasis, and therapeutic objectives, is indispensable.^[18]^

The management paradigm for mRCC is undergoing rapid evolution. Options such as cytoreductive nephrectomy (CN) and surgical metastasectomy remain viable for a subset of patients. Despite the established effectiveness of monotherapies, including targeted and immunotherapies, combination therapies have emerged as the standard of care in the treatment of mRCC. Ongoing research efforts are focused on refining therapy algorithms and identifying therapeutic approaches that offer reduced toxicity and enhanced efficacy. In the management of patients with mRCC, it is imperative for clinicians to consider a multitude of factors, such as the patient’s overall health status, tumor characteristics and stage, the feasibility of treatment, potential side effects and risks, and projected long-term outcomes, with the aim of formulating a tailored treatment plan that optimally aligns with the patient’s specific needs.

### 2.1. Surgical therapy

Surgical resection of metastases is widely recognized as a prevalent therapeutic approach for managing metastatic disease, particularly for individuals experiencing metastatic progression post-nephrectomy.^[19]^ According to the National Comprehensive Cancer Network (NCCN) pertaining to RCC, patients with oligometastatic recurrence subsequent to nephrectomy are considered eligible for metastasectomy under specific circumstances.^[20]^ The European Association of Urology recommends metastasectomy as a suitable local treatment for the majority of mRCC sites, with the exclusion of brain and bone metastases.^[21]^ Furthermore, the American Society of Clinical Oncology (ASCO) guidelines propose consideration of localized treatments directed at metastases solely for a select group of patients, notably those characterized by favorable risk factors as defined by the International Metastatic RCC Database Consortium (IMDC), suggesting that such interventions may represent the most advantageous strategy for these individuals.^[22]^ Additionally, research underscores the efficacy of CN, a surgical intervention aimed at removing primary renal neoplasms amidst metastatic disease, in enhancing survival rates among patients who exhibit minimal comorbid conditions, low surgical risks, and no metastases to the liver, brain, or bone, provided that at least 75% of the tumor mass is amenable to resection.^[23]^ An analysis conducted by Chen et al elucidates that in the management of mRCC, the employment of upfront CN alone, and in conjunction with targeted therapy, has been demonstrated to significantly enhance survival outcomes. However, the analysis further reveals no significant disparity in survival rates between patients receiving a combination of initial CN and immunotherapy and those undergoing immunotherapy followed by delayed CN.^[24]^ Nonetheless, in the contemporary landscape dominated by targeted and immunotherapy modalities, the integration of CN with systemic therapy remains the focal point of ongoing discourse, as specialists grapple with determining the strategy that most significantly augments patient longevity.

### 2.2. Targeted therapy

First-line targeted drugs for mRCC encompass a spectrum of inhibitors directed towards the VHL/HIF-1α/PDGF/VEGF pathway, including sunitinib, pazopanib, sorafenib, axitinib, cabozantinib, lenvatinib, bevacizumab, and belzutifan, in addition to agents that inhibit the mTOR pathway, such as everolimus and temsirolimus.^[25]^ These drugs are characterized by favorable toxicity profiles and improved efficacy.^[26]^ Concurrently, extensive research into molecular markers like CAIX, PTEN, and CXCR4, along with gene expression profiling, gene mutations, and methylation status has been conducted to refine treatment strategies of mRCC. Although these methodologies have not yet yielded significant improvements in augmenting current prognostic models, the intricate molecular characterizations of mRCC hold substantial promise in steering the development of tailored targeted therapeutic strategies.^[21]^

Cabozantinib, a multi-tyrosine kinase receptor inhibitor, has been the subject of analysis.Research conducted by Carsten Nieder et al revealed that patients with bone metastases from RCC (BOM-RCC) who were treated with cabozantinib demonstrated significantly improved prognoses when compared to those administered everolimus.^[27]^ In the CheckMate 9ER trials, cabozantinib in combination with nivolumab, as opposed to sunitinib monotherapy, resulted in extended OS (37.7 vs 34.2 months), prolonged PFS (16.6 vs 8.3 months), and a higher overall response rate (ORR) (55.7% vs 27.1%).^[28]^ Consequently, the combination of nivolumab and cabozantinib received FDA approval in January 2021. However, cabozantinib is moving between first and second line setting. Hirsch et al observed that cabozantinib demonstrated a generally favorable safety profile and exhibited intracranial efficacy in patients with brain metastases from RCC (BRM-RCC), irrespective of the line of therapy administered. Despite this, OS for a substantial proportion of patients with BRM-RCC remains constrained.^[29]^ Comparative studies on the efficacy of cabozantinib versus everolimus in second-line treatments for mRCC have shown that cabozantinib-treated patients experience longer OS (21.4 vs 16.5 months), a higher ORR (17% vs 3%), and improvements in PFS. However, the incidence of grade 3–4 adverse events was higher in the cabozantinib group compared to the everolimus group (71% vs 60%), indicating challenges in administering cabozantinib at its current dosage.^[30,31]^ While cabozantinib has demonstrated efficacy in managing mRCC, its effectiveness varies across the patient population. Therefore, it is imperative for clinicians to tailor treatment regimens with cabozantinib to the individual characteristics and conditions of each patient to optimize therapeutic outcomes and minimize adverse effects.

Belzutifan, a novel small molecule inhibitor targeting HIF-2α, has exhibited promising outcomes in various recent clinical investigations. This agent, recently authorized by the FDA for the management of non-mRCC, has also demonstrated significant therapeutic efficacy in the treatment of mRCC. Data from a phase 3 trial indicate that belzutifan significantly enhances PFS (HR 0.75, 95% CI 0.63–0.90, *P* < .001) and objective ORR (22% vs 3.5%), showing a higher frequency of complete responses compared to everolimus.^[32]^ In addition, objective remission rates were 25% and 22% in mRCC patients treated with belzutifan in combination with cabozantinib or levatinib, respectively.^[33]^ These outcomes suggest that belzutifan, both as a standalone treatment and in combination with other agents, offers considerable potential in addressing mRCC. Furthermore, the efficacy and tolerability of a triple-combination therapy involving pembrolizumab, lenvatinib, and belzutifan in a phase 3 trial for mRCC are currently under investigation, highlighting the ongoing exploration of belzutifan therapeutic scope.

### 2.3. ICIs therapy

Recent findings from specific clinical investigations, including the Keynote-564 trial^[34]^ and a phase 3 trial,^[35]^ have highlighted the encouraging potential of ICIs in the management of mRCC. Specifically, pembrolizumab, a notable ICI, has been shown to prolong disease-free survival (DFS) and has consequently been authorized for adjuvant use by the U.S. Food and Drug Administration. Choueiri et al documented that in RCC patients facing a heightened risk of disease recurrence, adjuvant treatment utilizing pembrolizumab surpasses placebo in terms of efficacy and maintains a tolerable safety profile without detriment to the quality of life.^[36]^ This position is currently endorsed by a consensus among experts, who further propose that a one-year pembrolizumab regimen should be established as the exclusive adjuvant therapeutic strategy.^[34,37]^ However, due to the rapid advancements within this domain, it is imperative to conduct ongoing evidence-based evaluations to stay abreast of emerging therapeutic options and insights.

### 2.4. Neoadjuvant therapy

Neoadjuvant therapy represents a robust and dynamic field of clinical investigation with ongoing exploration of its potential. The efficacy of neoadjuvant therapy with PD-1 monoclonal antibodies has been established. Additionally, a phase II study showed that administering neoadjuvant nivolumab prior to nephrectomy is both safe and feasible, without significant surgical delays or a high occurrence of immune-related adverse events.^[38]^ In select clinical cases, neoadjuvant targeted therapy has proven its clinical validity, enhancing the probability of employing conservative surgical treatment.^[39]^ Within the scope of combination therapies, the use of neoadjuvant sitravatinib plus nivolumab, despite not markedly improving the ORR, has been suggested to alter the immune microenvironment, thereby possibly increasing the likelihood of achieving a 24-month DFS in case of advanced RCC.^[40]^ Nonetheless, the definitive role and safety profile of neoadjuvant therapy in RCC will remain uncertain until data from phase III trials are made available.

### 2.5. Combination therapy

The therapeutic landscape for mRCC patients is rapidly changing as ICI–tyrosine kinase inhibitor (TKI) and ICI–ICI combinations demonstrated to improve oncological outcomes compared to standard of care.

In treatment-naïve mRCC patients, atezolizumab combined with bevacizumab surpassed sunitinib in terms of PFS, achieving a median PFS of 15.1 months versus 11.1 months in the IMmotion151 trial.^[41]^ The CheckMate 214 trial revealed that the combination of nivolumab and ipilimumab significantly improved OS in intermediate- and poor-risk mRCC patients compared to sunitinib, with an 18-month OS rate of 75% for nivolumab plus ipilimumab versus 60% for sunitinib.^[42]^ At ASCOGU 2024, Tannir et al reported the longest follow up for ICI-based combinations as first line treatment in mRCC. Their findings indicate that, relative to sunitinib, the combination of nivolumab and ipilimumab maintained a stable hazard ratio for OS over an 8-year period in intent-to-treat and intermediate/poor risk patients. PFS probabilities at 90 months were notably higher, ranging between 23% to 25%. Regardless of IMDC risk, responses were deep and durable, with longer duration and more complete responders. The long-term safety profile was deemed manageable, reinforcing the combination of nivolumab and ipilimumab as a standard of care for advanced RCC.

Additionally, in the CLEAR trial, the combination of ICI and TKI (lenvatinib and pembrolizumab) demonstrated superior outcomes compared to sunitinib monotherapy. This regimen exhibited a notable complete response rate of 16.1% and an ORR of 71%, alongside significant enhancements in PFS and OS.^[43]^ Phase 3 studies conducted by Viktor Grünwald et al substantiated the augmented efficacy of pembrolizumab combined with lenvatinib versus sunitinib in patients with RCC-irrespective of the presence or absence of baseline lung, bone or liver metastases, prior nephrectomy, or sarcomatoid features, thereby underscoring the robustness of this therapeutic approach in managing RCC.^[44]^ The KEYNOTE-426 trial demonstrated that pembrolizumab plus axitinib outperformed sunitinib monotherapy in previously untreated advanced RCC, with a 12-month OS rate of 89.9% versus 78.3%.^[45]^ A 4-year follow-up of a phase Ib trial revealed that a majority (73.1%) of patients administered with the combination of pembrolizumab and axitinib are still alive, without emergence of new safety concerns. The long-term results further bolster the endorsement of pembrolizumab plus axitinib as a first line therapeutic strategy for patients with advanced RCC.^[46]^ It is evident that, compared to monotherapy, the integration of ICI with existing TKI is emerging as an potent strategy capable of enhancing survival outcomes in mRCC. However, the decision to employ ICI in combination as adjuvants therapy outside of clinical trials should be based on the specific pathology of the tumor tissue, and the optimal regimen for combining ICI necessitates ongoing exploration in clinical trials.

Furthermore, the researchers conducted a comparative analysis of the efficacy between the ICI + ICI and ICI + TKI. Benedikt Hoeh et al reported that no significant differences in PFS or OS were identified between the ICI + ICI (nivolumab + ipilimumab) versus ICI + TKI (pembrolizumab + axitinib) treatment regimens in the overall cohort.^[47]^ However, another study indicated that the ICI combination was associated with longer PFS and time to next treatment than TKIs monotherapy. Among ICI combinations, ICI + TKI was associated with significantly improved PFS and time to next treatment compared to ICI + ICI.^[48]^

In summary, doublet combination therapies have yielded significantly enhanced clinical outcomes and are established as the standard of care, involving either 2 ICIs or a combination of an ICI and TKI. Trials investigating triplet combination therapies are being conducted, albeit with the caveat of increased toxicity. To optimize the therapeutic index by balancing efficacy and toxicity, extensive research is underway exploring various combination treatments. This endeavor aims to broaden the spectrum of first-line treatment alternatives in the future.

## 3. The site of RCC metastasis

Patients with RCC may develop metastatic disease at various anatomical sites. Synthesizing evidence from several researches conducted by Chatzizacharias NA et al, Komiyama T et al, Dudani S et al, and a study of IMDC,^[49–52]^ we suggest that despite disparities in research outcomes regarding the common sites for RCC metastasis and discrepancies in the incidence of metastasis to different organs among distinct subtypes of RCC (Clear Cell RCC [ccRCC], papillary RCC, and chromophobe RCC), the predominant sites of metastasis can be delineated as follows: lungs, lymph nodes (LNs), bones, liver, brain, and adrenal glands. Notably, Gen’an Longren et al found that the therapeutic efficacy in mRCC may diverge contingent upon the specific organ site of metastasis.^[53]^ Therefore, it is necessary to understand the therapeutic and prognostic distinctions tailored to various organ-specific metastatic manifestations of RCC.

### 3.1. Lung metastasis from RCC (LM-RCC)

**Figure 2.**
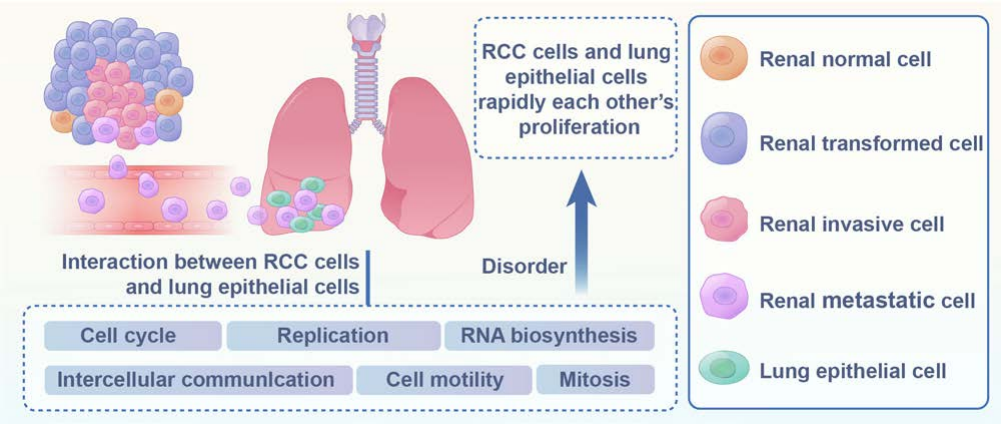
The mechanism of lung metastasis in RCC. RCC = renal cell carcinoma.

The lung is the predominant site for RCC metastasis, accounting for 43.6% of metastatic occurrences in kidney cancer.^[54]^ LM-RCC is commonly manifested as either single or multiple pulmonary nodules, where single nodules are at times misdiagnosed as primary lung carcinomas in clinical environments.^[55]^ Research has indicated that the latency of lung metastasis is markedly shorter than that of the growth period, implying that early identification of the primary neoplasm does not prevent lung metastasis.^[56]^ Katarzyna Kaminska et al have elucidated the underlying mechanisms of LM-RCC, proposing that the interaction between RCC cells and pulmonary epithelial cells not only stimulate the proliferation of lung epithelial cells but also the rapid expansion of RCC cells. This cross-talk between RCC and normal lung cells disrupts gene expression, affecting various signaling pathways related to cell cycle, DNA replication, cell motility, and RNA biosynthesis (Fig. 2).^[57]^ These pathways are considered “druggable” for therapeutic interventions, offering avenues to treat mRCC and improve clinical outcomes.

Zhang et al have identified independent prognostic factors for patients with lung metastasis from ccRCC, including sex, age, tumor grade, N stage, T stage, and the presence or absence of bone or brain metastases. However, these factors may not be applicable to all patients.^[58]^ Furthermore, Xinyu Sheng et al have developed a nomogram based on the SEER database to predict the prognosis for patients with LM-RCC. This model incorporates 7 factors, including pathological type and clinical and treatment characteristics, providing direct insight into patient survival rates.^[59]^

In general, surgical outcomes for LM-RCC are superior.^[60]^ Studies have shown that survival benefits offer by an aggressive surgical eradication of LM-RCC, with 5-year survival rates reported to range from 18% to 75%.^[61]^ It is noteworthy that complete resection is a significant predictor of a favorable prognosis and can extend survival by delaying the initiation of immunotherapy or targeted therapy or enabling complete response after these therapies.^[62,63]^ This perspective is reinforced by Ueno et al, who advocate for the performance of pulmonary metastasectomy in RCC patients harboring sarcomatous features, following the attainment of either. partial or complete remission via initial systemic therapy.^[64]^ Further, Stewart et al have identified radiation therapy as a viable method for managing the prevalent incidence of LM-RCC, effectively addressing lesions that constitute significant mortality threats.^[65]^ Contrarily, Gonnet et al present radiofrequency ablation (RFA) as a secure and efficacious surgical substitute within an integrated systemic treatment strategy, with patients undergoing RFA for LM-RCC exhibiting favorable OS and prolonged systemic treatment-free survival.^[60]^

Consequently, resection of metastatic pulmonary lesions, especially when a complete resection is viable, continues to be the foundational approach in the treatment of LM-RCC. RFA can serve as a suitable alternative to surgical resection when patients enter the systemic treatment phase. However, the optimal timing for performing metastasectomy, with or without systemic treatment, necessitates further investigative efforts.

### 3.2. LN metastasis from RCC (LNM-RCC)

Traditionally, lymphatic vessels were perceived as mere pathways facilitating the invasion of tumor cells into adjacent tissues during the metastatic process. However, recent advancements in research have unequivocally demonstrated that both the neogenesis of lymphatic vessels and remodeling of preexisting ones are crucial contributors to the metastatic spread of tumors. Tumor cells can infiltrate the lymphatic system either through the invasion of preexisting lymphatic vessels in the surrounding tissues or by prompting the formation of new lymphatic vessels within the tumor itself.^[66,67]^ In the context of LNM-RCC, there exists pronounced variability in the patterns of invasion across different lymphatic regions, including the paracaval, precaval, interaortocaval, and para-aortic LN.^[68]^ This heterogeneity underscores the complexity of LNM-RCC, highlighting the need to understand lymphatic involvement in the metastatic pathway.

LN involvement in RCC is associated with poor prognosis, and the presence of LN metastases constitutes a worse prognostic factor in RCC.^[69,70]^ Zareba et al revealed that a higher number of positive LNs correlates with higher all-cause mortality, while a greater quantity of negative LNs was associated with improved OS.^[71]^ In a retrospective study focusing on the predictive value of positive LNs for OS in RCC patients established that the number of positive LN, LN ratio, and log odds of positive LN (LODDS) are independent prognostic factors for OS. It was observed that LN ratio and LODS predictive performance that is both comparable to each other and superior to that of the number of positive LN. Specifically, the LODS metric displayed enhanced effectiveness in distinguishing survival outcomes among RCC patients.^[72]^ Furthermore, a novel predictive model developed by Sun et al incorporates low age, large LN size on preoperative imaging, and high clinical T stage as independent predictors of pathologic LN metastasis in RCC patients. This model shows potential for the more precise presurgical prediction of LNM-RCC, thereby enabling earlier clinical interventions and potentially improving patient prognosis.^[73]^

Lymph node dissection (LND) has been proposed as a strategy to improve the outcomes of surgical therapy for many genitourinary malignancies. Nonetheless, the therapeutic efficacy of LND in RCC remains a subject of debate.^[74]^ Research by Blom et al indicated no substantial differences in OS, PFS, or time to disease progression between patients undergoing comprehensive LND in conjunction with radical nephrectomy and those receiving radical nephrectomy alone.^[75]^ A retrospective analysis similarly found no survival advantage, the 5-year OS rates for patients undergoing LND were 21%, compared to 31% for those who did not, without a significant difference.^[76]^ Gershman et al conducted a study on 305 patients with mRCC and found that LND at the time of CN did not confer a survival advantage, even for patients with a higher risk of positive LNs.^[77]^ However, Capitanio et al revealed a survival benefit from LND in a subset of RCC patients with locally advanced disease and/or unfavorable clinical and pathological characteristics (higher tumor stage, grade, or the presence of sarcomatoid features).^[78]^ There is also evidence to suggest that LND combined with CN may improve survival rates prior to the initiation of systemic immunotherapy.^[69]^ Furthermore, Rosiello et al emphasized that an extensive LND could be justified in patients with large suspicious LN metastases.^[79]^ Daniel et al suggested the reclassification of RCC with isolated LN metastases to stage IV to enable more precise baseline risk stratification for patients with LNM-RCC.^[80]^ Given the current lack of robust and consistent evidence from retrospective studies on LND, further research is imperative to evaluate the efficacy of LND and to improve the stratification of patients, identifying those who may derive benefit from the intervention.

### 3.3. BOM-RCC

In the context of RCC, the bone represents the predominant site for hematogenous dissemination of tumor cells.^[81]^ Bone metastases occur in about 20% to 35% of patients with mRCC, and these patients have a 5-year survival rate of <11%.^[82]^ Predominantly osteolytic, these bone metastases lead to extensive skeletal destruction. Consequently, many patients with RCC experience skeletal-related events (SREs), such as pain, fractures, and nerve compression, particularly in advanced disease stage.^[83]^ While selecting patients with BOM-RCC can achieve a cure, the prognosis is typically poor. Therefore, the principal treatment objectives are to alleviate pain and prevent or delay SREs in these patients.^[84]^

**Figure 3.**
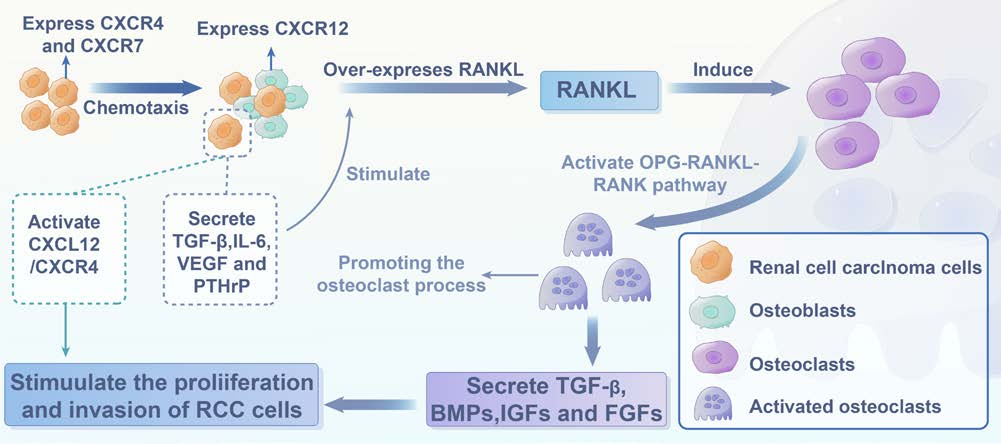
The mechanism in the development of bone metastasis in renal cell carcinoma.

The “vicious circle” hypothesis posits a reciprocal relationship between cancer cells and the bone microenvironment that catalyzes both tumor growth and bone destruction, a concept that is widely accepted as the primary mechanism driving BOM-RCC.^[85]^ Research has shown that cancer cells possess a strong chemotactic response osteoblasts, which secrete chemokine family members such as CXCL12. These cancer cells, in turn, secrete chemokine receptors such as CXCR4 and CXCR7. CXCL12/CXCR4 activation significantly enhances the proliferation and invasion of cancer cells.^[86]^ Furthermore, cancer cells release various factors including TGF-β, IL-6, VEGF, and PTHrP, which stimulate osteoblasts to increase RANKL expression. The upregulation of RANKL induces the activation and growth of osteoclasts through the osteoprotegerin-RANK pathway, thereby promoting the osteoclastic activity of the cancer cells.^[87]^ Concurrently, these osteoclasts resorb bone and secrete growth factors such as BMPs, IGFs, and FGFs, which further stimulate the proliferation of metastatic cancer cells^[88]^ (Fig. 3). Thus, targeting the mechanisms underpinning the “vicious circle” hypothesis could herald a novel direction in the treatment of BOM-RCC.

Prognostic factors have been extensively explored in patients with BOM-RCC. Santoni et al identified critical prognostic factors for patients with BOM-RCC, including age, ECOG physical status (ECOG PS), histology, MSKCC prognosis score, presence of concomitant metastases, and the interval between nephrectomy and the onset of bone metastasis.^[89]^ Singh et al have proposed that alkaline phosphatase, C-reactive protein, hemoglobin levels, and erythrocyte sedimentation rate serve as potential risk factors for both concurrent bone metastasis in advanced RCC and the emergence of SREs.^[90]^ Additionally, poor prognosis is strongly correlated with liver involvement, more than one bone metastasis, and the absence of nephrectomy.^[91]^ While radical bone surgery may extend survival for a subset RCC patients, the overall prognosis for many is inextricably linked to SRE features. Hypercalcemia, in particular, is identified as a particularly poor prognostic indicator. Notably, neither the timing of bone metastasis nor the incidence of fractures was found to have a significant impact on prognosis.^[92]^ It can be seen that a unified consensus on standardized prognostic indicators remains elusive.

Patients exhibiting a single or a limited quantity of resectable metastases are deemed appropriate candidates for comprehensive surgical resection. The integration of surgery with targeted therapy emerges as a strategic approach for achieving therapeutic effects, attaining local tumor control, and enhancing survival rate.^[93]^ Furthermore, stereotactic body radiotherapy has also been proposed as a safe and effective treatment for patients with BOM-RCC harboring up to 5 lesions. Nonetheless, after 13 months of metastasis-directed localized therapy, a substantial fraction of these patients, nearly half, exhibit disease progression, particularly manifesting as distant metastasis beyond the initially treated sites.^[94,95]^ For patients with multiple bone metastases, a synergistic approach incorporating both local and systemic treatments is recommended.^[96]^ Currently, denosumab and zoledronic acid are recognized as the primary systemic pharmacological treatments for BOM-RCC. Research indicates that denosumab significantly prolongs the interval to the first SREs in patients with bone metastasis from solid tumors and markedly diminishes the cumulative incidence of SREs in comparison to bisphosphonates.^[97]^ An analysis by CARSTEN NIEDER et al demonstrated that the patients with BOM-RCC undergoing treatment with cabozantinib exhibited significantly improved prognoses compared to those receiving everolimus. Nonetheless, the OS for a vast majority of patients continues to be restricted.^[98]^ Recent advancements in image-guided ablation technologies have shown considerable promise in managing pain associated with osseous metastases.^[27]^ In the study by Gardner et al, minimally invasive cryoablation could represent a viable treatment alternative for approximately 75% of BOM-RCC patients who are not candidates for surgical resection. However, this technology is not appropriate for individuals with lesions in weight-bearing bones of the appendicular skeleton, especially those with an elevated fracture risk, where surgical intervention remains the preferred option.^[99]^ Microwave ablation (MWA) has emerged as a novel alternative therapy, effectively alleviates pain and improving functional outcomes.^[27]^ Zheng et al have reported that a conservative surgery complements by either percutaneous or intraoperative MWA presents a viable and safe option for select patients with recurrent bone tumors in the limbs.^[100]^ Furthermore, Jin Ke et al have discovered that image-guided percutaneous thermal MWA for addressing bone metastases significantly reduces pain and improves mobility, with CT imaging being the optimal modality for guiding percutaneous ablation procedures.^[101]^ Consequently, different therapeutic strategies have demonstrated distinct therapeutic advantages. It is recommended to adopt a personalized treatment methodology, meticulously customized to the unique clinical presentations and characteristics of each patient.

### 3.4. Liver metastasis from RCC (LVM-RCC)

Relative to RCC metastasizing to the lungs, LNs, and bones, liver metastasis is relatively infrequent. Nevertheless, patients with LVM-RCC exhibit an exceedingly unfavorable prognosis characterized by a shorter median OS.^[102]^ The underlying reasons for the poor prognosis remain elusive in patients with LVM-RCC. A research indicates a potential link with the unique hepatic microenvironment and liver dysfunction in patients with metastasis.^[103]^ Moreover, conducting large-scale cohort or randomized prospective studies concerning LVM-RCC is challenging owing to its rarity, unclear mechanisms, and the absence of established clinical guidelines.^[104]^

The existing literature concerning hepatic resection for RCC metastases remains limited, with the majority of studies on LVM-RCC concentrating on the evaluation of diverse clinical therapeutic strategies. For instance, in the largest study to date exploring the role of CN in LVM-RCC, it was observed that CN could significantly enhance the survival rates in carefully selected patients with LVM-RCC.^[105]^ While the importance of systemic therapy continues to grow, liver metastasectomy remains a valuable independent treatment option for LVM-RCC patients and can markedly improve OS if technically feasible.^[106]^ This view is corroborated by Anthony T Ruys et al and Chatzizacharias et al who advocate for the application of liver metastasectomy in LVM-RCC, particularly in cases where negative margins can be achieved through surgical resection.^[107]^ Additionally, HAMADA et al affirm the potential benefits of localized liver therapeutics, such as surgical excision for LVM-RCC patients characterized by limited metastatic locations, a minimal number of metastases, and favorable ECOG PS. For other LVM-RCC cases, treatment with TKIs may offer benefit. However, LVM-RCC patients with an ECOG PS ≥ 2 typically exhibit a poor prognosis, irrespective of local or systemic treatment.^[108]^

Furthermore, advancements in image-guided ablation techniques have facilitated the development of efficacious local treatment for liver metastases.^[109]^ Studies have demonstrated that MWA and RFA are recognized as both safe and efficient modalities for the management of liver metastases.^[110]^ And, within the context of LVM-RCC, MWA has been indicated to offer several practical benefits over other techniques, including a larger ablation volume, shorter surgical time, and a reduced heat-sink effect, without an increase in recurrence rate.^[111]^ Moreover, the study by Peng et al has shown that the integration of sunitinib with MWA effectively prevents the progression of unresectable LVM-RCC,^[112]^ suggesting that the combination of targeted pharmacotherapy with ablation technology holds potential for novel therapeutic avenues.

Certainly, some studies have been conducted on the prognostic predictors of LVM-RCC. The study by Sung Han Kim et al‘s highlighted that liver metastasis in conjunction with unfavorable Heng risk factors was a significant risk factor for poor PFS and OS in patients with mRCC undergoing systemic therapy. Fleckenstein et al have established the utility of 3D tumor quantification analysis as a reliable predictive indicator for OS in the context of assessing the efficacy of intra-arterial therapy for patients with LVM-RCC.

It is essential to acknowledge that LVM-RCC is associated with the most unfavorable prognosis among all metastatic sites, characterized by limited medium- and long-term follow-up data and the shortest survival times, underscoring the paucity of evidence for LVM-RCC management. In clinical practice, it is imperative to conduct comprehensive patient assessments and proactively seek alternative treatment strategies.

### 3.5. BRM-RCC

BRM-RCC is often characterized by worse bleeding and vascular edema. Brain metastasis is a relatively common complication in patients with advanced RCC.^[113,114]^ Generally, chromophobe RCC exhibits a low propensity for brain metastasis, whereas ccRCC is notably more prone to such metastatic spread. Nonetheless, the prognosis patients with BRM-RCC is exceptionally poor across different cellular subtypes,^[115]^ with the average survival time post-diagnosis not exceeding 1 year.^[116]^ The adverse prognosis associated with BRM-RCC has been attributed to several factors, including its persistent resistance to radiotherapy, frequent emergence of symptomatic neurological dysfunction, and limited permeability of many drugs through the blood-brain barrier.^[117–119]^ Currently, recognized prognostic factors for patients with BRM-RCC include the number of brain metastases, the presence of a sarcomatoid component, extracranial metastasis, and Karnofsky Performance Status.^[120,121]^

**Figure 4.**
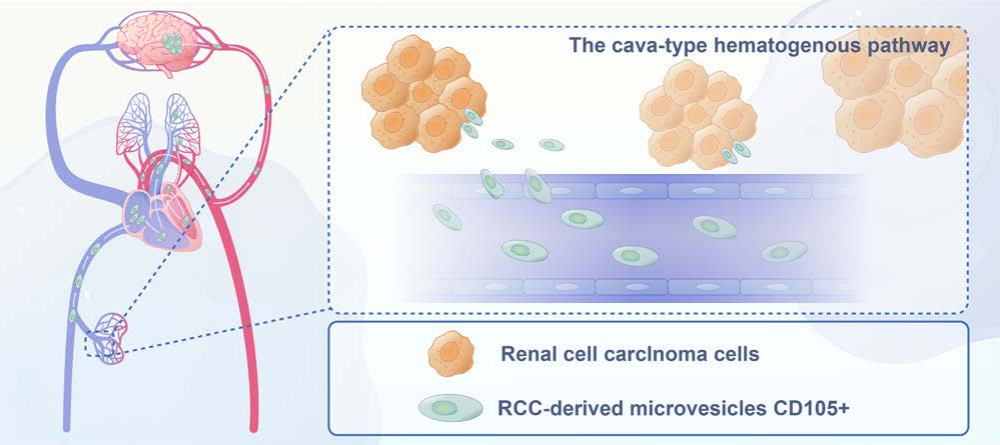
The cava-pathway and subsequent steps in the development of brain metastasis in renal cell carcinoma.

BRM-RCC frequently occurs as a metachronous dissemination via several pathways, with the cava-type pathway being the most prevalent, representing approximately 75% of cases. In these cases, metastatic dissemination is believed to commence with the introduction of CD105 + microbubbles originating from RCC into the bloodstream. These microbubbles, which differ from the cells at the primary tumor site, share phenotypic characteristics with tumor stem cells and have the capacity to induce angiogenesis. They traverse the right ventricle to enter the pulmonary capillaries and arteries, eventually reaching the cerebrovascular system.^[120]^ The infiltration of tumor cells into the brain is facilitated by an increase in vascular permeability and disruption of the blood-brain barrier (Fig. 4)^[122]^

The brain, a vital organ with restricted regenerative abilities post-injury, poses significant treatment challenges in cases of BRM-RCC. The selection of an optimal treatment strategy necessitates a thorough evaluation of multiple factors, including the potential long-term toxicity, projected patient survival, and the presence of competing risks.^[123–125]^ Currently, the consensus within the BRM-RCC therapeutic landscape favors neurosurgery or stereotactic radiosurgery (SRS) as the gold standard for single or oligometastases.^[120,126]^ SRS, in particular, has been recognized for its capacity to effectively manage cerebral manifestations of BRM-RCC, achieving diseases control in most cases with fewer complications, thereby enhancing the quality of survival, especially when brain metastases are identified early.^[127]^ The evidence further suggests that administering a single preoperative SRS significantly diminishes the risk of radiation necrosis (RN) or leptomeningeal disease, in contrast to postoperative SRS. Although instances of subtotal resection are rare, they are closely linked to worse outcomes regarding RN, leptomeningeal disease, and OS.^[128]^

In addition, emerging treatments employing immunotherapeutic or targeted agents have shown potential in improving prognoses for BRM-RCC patients. Notably, Nivozumab, characterized by its favorable tolerance profile and minimal unexpected toxicity, exhibits limited efficacy in patients with BRM-RCC in the absence of local treatment.^[129]^ Hirsch et al proposed that cabozantinib, a multi-tyrosine kinase receptor inhibitor, is generally safe and displays intracranial activity, albeit with poor prognosis.^[29]^ A phase 3b/4 study have revealed that the combination of ipilimumab and nivolumab in immunotherapy is safely administered, showing promise in efficacy despite poor prognosis and significant unaddressed medical needs.^[130]^ Nevertheless, due to the scarcity of evidence, a consensus regarding the effectiveness and activity of these treatments remains unachieved within the scientific community.

Recent advocacy by researchers have focused on the development of diverse therapeutic strategies that incorporate a range of treatment modalities alongside SRS, guided by clinical characteristics.^[131]^ Their findings suggest that the inclusion of SRS as a fundamental component in the management of BRM-RCC significantly reduces the mortality related to brain dysfunction and enhanced functional brain activity.^[131]^ Furthermore, an international multicenter study conducted by Lehrer et al, established that the concurrent use of ICIs with SRS in the treatment of BRM-RCC patients was found to be safe, with a minimal incidence of symptomatic RN.^[132]^ Uezono similarly concluded that BRM-RCC patients receiving SRS in conjunction with ICIs experienced prolonged survival, emphasizing the effectiveness and benefits of reducing the SRS dose.^[133]^ Some researchers have proposed that a combination of systemic therapy and localized treatments can extend the survival time of BRM-RCC patients, advocating for an aggressive and continuous multimodal therapy.^[134,135]^ The paramount goal presently remains the improvement of BRM-RCC patient survival rates. Following the clinical identification of BRM-RCC, it is essential to devise a tailored multimodal treatment plan, taking into consideration the unique characteristics of each patient. Therefore, there exists an imperative to establish exhaustive guidelines for the management of BRM-RCC.

### 3.6. Adrenal gland metastasis from RCC (AGM-RCC)

The adrenal gland is a common site for metastasis in various malignant neoplasms, with a significant correlation observed in patients with a history of RCC, thereby considing it as a potent risk factor.^[136]^ The mechanisms of AGM-RCC include direct invasion, lymphovascular spread, and retrograde venous embolization.^[137]^ The incidence of AGM-RCC is documented at 3% to 5% for ipsilateral adrenal gland metastasis from RCC, approximately 0.7% for contralateral adrenal gland metastasis from RCC, and a notably rare occurrence for bilateral adrenal gland metastasis from RCC (BAGM-RCC).^[137]^ The determination of the most effective diagnostic strategy for adrenal tumors in patients with a history of RCC is still under debate. Contrast-enhanced CT scans are favored for diagnosing adrenal metastases, however, relying solely on radiological assessment may not suffice to distinguish between primary adrenal neoplasms, adrenocortical adenomas, and metastatic lesions in patients with RCC.^[136,138]^ Research indicates that AGM-RCC metastatic tumors often possess a vascular nature, which sets them apart from the more hypovascular adrenal cortical adenomas or carcinomas. In this context, adrenal mass biopsy could be instrumental in the diagnostic evaluation of patients with suspected metastases.^[139]^

There are reports suggesting a decline in adrenal gland function after ipsilateral adrenal involvement. Patients with ipsilateral adrenal gland metastasis from RCC still have a challenging prognosis despite complete resection.^[140]^ Given this unfavorable prognosis, Moudouni et al have advocated for the classification of adrenal involvement as a distinct category within staging systems.^[141]^ However, a study suggest that adrenalectomy is deemed unnecessary for patients with early RCC to a limited extent, kidney surgery for a renal mass should be performed only if radiographic or intraoperative evidence indicates adrenal gland involvement, including removal of the ipsilateral adrenal gland.^[142]^ On the other hand, KESSLER et al argued that in cases where the average interval between nephrectomy and the emergence of adrenal metastases exceeds 18 months, the adrenal gland removal is advised as it is expected that a subset of these patients are anticipated to achieve long-term survival.^[143]^ Nevertheless, when systemic adjuvant therapy becomes available for RCC, the risks and benefits of adrenalectomy combined with radical nephrectomy should be reassessed.^[141]^

While contralateral adrenalectomy may offer advantages to certain patients with contralateral adrenal gland metastasis from RCC, yet there is a notable lack of comprehensive data on the surgical outcomes.^[144]^ Encouragingly, patients with a solitary metastasis to the contralateral adrenal gland typically do not exhibit symptoms or adrenal dysfunction post- nephrectomy.^[144,145]^ Recently, Li et al corroborated that surgical intervention is an appropriate treatment modality for BAGM-RCC patients, resulting in improved survival rates.^[146]^ However, there is no standardized approach in the literature for treating patients with BAGM-RCC, with metastasectomy emerging as the most viable therapeutic option.^[147]^

Arriving at a balanced conclusion is rendered difficult by the variability in study designs and the heterogeneity of data. The treatment strategies for ipsilateral adrenal gland involvement necessitate a tailored assessment of the surgical risks versus benefits for each patient. Currently, due to the scarcity of data on alternative therapeutic approaches, metastasectomy stands as the most feasible treatment strategy for both contralateral and bilateral adrenal gland metastases.

## 4. Conclusion

Presently, neither the NCCN nor EAU guidelines provide detailed insights into the variations in the treatment and prognosis of RCC metastases to specific organs. Understanding the distinctions in the stratification of RCC metastases to individual organs can facilitate tailored treatment in clinical patients. Some organs, such as the lungs, LNs, and pancreas, often yield favorable outcomes following surgical intervention or pharmacotherapy. However, liver and brain metastases typically have a poor prognosis, regardless of the treatment strategy. Bone metastases represent an intermediate category, characterized by a moderate prognosis. The surgical approaches for brain and adrenal gland metastases tend to be relatively complex. SRS can offer considerable relief from the local symptoms of both brain and bone metastases. The identification of metastatic sites not only provides prognostic value but also plays a critical role in guiding clinical treatment decisions. Nonetheless, current research evidence remains insufficient, and prospective trials are necessary in many areas. The future of mRCC treatment is expected to revolve around multidisciplinary management.

## Acknowledgments

Sincere thanks for all support.

## Author contributions

**Conceptualization:** Guobin Weng.

**Data curation:** Zengxing Qian, Dongying Cheng, Lingmin Song.

**Funding acquisition:** Xue Wang, Junjun Wei, Xiaodong Chen.

**Project administration:** Guobin Weng.

**Writing – original draft:** Xue Wang, Lin Qian, Qihang Wu.

**Writing – review & editing:** Shuaihuai Huang, Ping Wang.
